# Derived asymmetric and transitive relations using the go/no‐go procedure with compound stimuli

**DOI:** 10.1002/jeab.70088

**Published:** 2026-02-01

**Authors:** Rafael Diego Modenesi, Paula Debert

**Affiliations:** ^1^ Universidade de São Paulo São Paulo Brazil; ^2^ Instituto Nacional de Ciência e Tecnologia sobre Comportamento Cognição e Ensino (INCT‐ECCE) Brazil

**Keywords:** compound stimuli, contextual cues, derived relations, go/no‐go, human, mouse‐clicking

## Abstract

The purpose of the present study was to verify whether the go/no‐go procedure with compound stimuli would establish derived asymmetric and transitive relations between stimuli under the control of contextual cues. In Experiment 1, nonarbitrary relational training and tests established red and blue background colors as the contextual cues. Subsequently, arbitrary relational training established relations between pairs of stimuli under the control of the contextual cues. Finally, tests evaluated the emergence of new relations under contextual control. All four participants, university students, met the learning criterion during training and demonstrated the derived relations that were tested. Experiment 2 replicated Experiment 1, without the nonarbitrary relational training and testing. All three participants also exhibited the derived performances. The effectiveness of the procedure for establishing derived relations, the implications of the necessity of nonarbitrary relational training, and the possibilities for application are discussed.

First demonstrated by Sidman (1971), new arbitrary emergent conditional relations were derived after directly training other arbitrary conditional relations with the matching‐to‐sample procedure (MTS). For example, training A‐B and B‐C conditional relations produces the emergence of B‐A, C‐B, A‐C, and C‐A relations. According to Sidman (1994), these emergent relations attest to the establishment of an equivalence class, and stimuli within this class are substitutable for each other.

Other types of derived relational responding have also been demonstrated experimentally, including opposition relations (e.g., Barnes‐Holmes et al., 2004; Belisle, Paliliunas, et al., 2023; Perez et al., 2015; Sbrocco et al., 2025; Steele & Hayes, 1991), comparative relations (e.g., Berens & Hayes, 2007; Dymond & Barnes, 1995; Luoma et al., 2024; Munnelly et al., 2013, 2019; Whelan et al., 2006), distinctive and categorial relations (e.g., Belisle, Lang, et al., 2023), hierarchical relations (e.g., Slattery & Stewart, 2014; Stewart et al., 2017), deictic relations (e.g., Rendón et al., 2012), and temporal relations (e.g., Brassil et al., 2019), among others (e.g., Hayes et al., 2001). These types of relations differ from equivalence relations in terms of the patterns of relational responding that are trained and that emerge without direct training. For example, opposition relations are typically characterized as symmetrical (i.e., if A is the opposite of B, then B is the opposite of A), nontransitive (i.e., if A is the opposite of B and B is the opposite of C, then A is not necessarily the opposite of C), and irreflexive (i.e., A is not the opposite of A). In contrast, comparative, deictic, and temporal relations are typically asymmetrical (i.e., if A is larger than B, then B is smaller than A), transitive (i.e., if A is larger than B and B is larger than C, then A is larger than C), and irreflexive (i.e., A is not larger than A).

Relational frame theory (RFT) conceptualizes the main properties of derived relational responding under the broader categories of mutual entailment, combinatorial entailment, and transformation of stimulus function. *Mutual entailment* refers to the bidirectional nature of relational responding, encompassing both symmetrical and asymmetrical relations between stimuli. *Combinatorial entailment* involves the coordination of two or more mutually entailed relations, resulting in relational networks that include both transitive and nontransitive patterns. A third property, *transformation of stimulus function*, refers to changes in the functions of a stimulus based on its derived relations with another stimulus (e.g., Hayes et al., 2001). Thus, the main characteristics that distinguish different types of relational responding involve the combination of properties such as symmetry, asymmetry, transitivity, and nontransitivity.

The present study focuses on asymmetrical and transitive relations such as comparative, deictic, ordinal, and temporal relations. Studies have frequently used the MTS procedure to establish these derived relations formats (e.g., Belisle et al., 2020; Dymond & Barnes, 1995; O'Hora et al., 2002; Perez et al., 2015; Roche & Dymond, 2008; Steele & Hayes, 1991; Whelan et al., 2006). Experiments usually begin with nonarbitrary relational training and testing in which conditional discriminations between physically similar stimuli are placed under the control of contextual cues. For example, during nonarbitrary training, Whelan et al. (2006) used the MTS procedure with a contextual cue as the sample (the words LESS‐THAN or MORE‐THAN) and two comparison stimuli (e.g., a figure of one apple and seven apples). When the contextual cue was LESS‐THAN, selecting the comparison stimulus with the smaller quantity (e.g., one apple) was followed by reinforcement. In the presence of MORE‐THAN, selecting the comparison stimulus with the larger quantity (e.g., seven apples) was followed by reinforcement. This training was performed with multiple exemplars (eight different figures, such as basketballs, beakers, etc., presented in different quantities). In subsequent tests, new figures were presented in the same manner, without reinforcement, to assess whether the contextual cues acquired control over the responses to the new nonarbitrary relations. In the second phase (arbitrary relational training), conditional discriminations between physically dissimilar stimuli were placed under the control of the contextual cues from the previous training. For example, in the study by Whelan et al. (2006), seven dissimilar stimuli (meaningless syllables such as “ZID” and “VEK”), referred to as A, B, C, D, E, F, and G, were presented with the contextual cue used in the previous phases. When the contextual cue was LESS‐THAN, the conditional relations AB, BC, and CD were taught. When the contextual cue was MORE‐THAN, the conditional relations ED, FE, and GF were taught. Finally, extinction tests evaluated the emergence of 36 new stimuli relations (e.g., LESS‐THAN [AC], LESS‐THAN [AG], MORE‐THAN [FB], MORE‐THAN [GE], and so on). The derived arbitrary relational responding detected in these tests comprising new relations indicated that the new relations between physically dissimilar stimuli were established without directly training them.

In addition to MTS, studies addressing derived relational responding have employed different procedures including successive go/no‐go (e.g., Brassil et al., 2019) and yes/no procedures (e.g., Sbrocco et al., 2025), the implicit relational assessment procedure (for a review, see Barnes‐Holmes & Harte, 2022), and the relational evaluation procedure (e.g., Munnelly et al., 2013). Another alternative to the MTS procedure is the go/no‐go procedure with compound stimuli, which has been widely used to establish equivalence relations (e.g., Gueiros & Debert, 2020; Canovas et al., 2019; Debert et al., 2007, 2009; Grisante et al., 2013; Modenesi et al., 2021) as well as to establish contextual control over equivalence classes (e.g., Modenesi & Debert, 2015). However, this procedure has not yet been implemented to verify the establishment of other derived relations.

The go/no‐go procedure with compound stimuli presents compound stimuli (pairs of stimuli presented side by side) successively for 4 s each. In each 4‐s trial, the participants can respond by clicking with the mouse (go responses) or refrain from responding (no‐go response) in the presence of each compound. In Debert et al.'s (2007) study with six undergraduate students, during the training phase, reinforcing consequences followed go responses when specific compound stimuli (A1B1, A2B2, A3B3, B1C1, B2C2, and B3C3) were present, whereas no consequences followed go responses in the presence of other compound stimuli (A1B2, A1B3, A2B1, A2B3, A3B1, A3B2, B1C2, B1C3, B2C1, B2C3, B3C1, and B3C2). Once accurate performances were established, extinction tests were carried out using compound stimuli BA/CB (symmetry test) and AC/CA (transitivity and equivalence tests). Five participants demonstrated emergent performance consistent with the formation of three equivalence classes (A1B1C1, A2B2C2, and A3B3C3).

As suggested by Debert et al. (2007), the go/no‐go procedure with compound stimuli may have certain benefits relative to the MTS procedure. For instance, the stimuli to be observed are closely spaced or presented as figure–ground (e.g., Debert et al., 2009) and thus can be scanned with little or no change in the direction of gaze. This feature of the go/no‐go procedure with compound stimuli more closely resembles real‐life experiences. As suggested by Munnelly et al. (2013), MTS, as a “top‐down” method of presenting the contextual cue above comparison stimuli, does not reflect the order in which individuals encounter relational stimuli in their everyday environment. Therefore, the go/no‐go procedure with compound stimuli may benefit populations who present difficulties in establishing emergent repertoires with MTS, such as children with autism spectrum disorder (ASD) who are at risk of developing restricted stimulus control (e.g., Stromer et al., 1993). For example, establishing conditional discriminations using MTS procedures may be difficult for some individuals with ASD because they show location bias (e.g., Bourret et al., 2012; Da Hora et al., 2018). The go/no‐go procedure with compound stimuli can be a good alternative, considering that stimuli to be related are presented side by side and it requires participants to respond—or refrain from responding—based on the relations between those stimuli. Besides that, the go/no‐go procedure was effective in establishing contextual control over equivalence classes (e.g., Modenesi & Debert, 2015) that would be necessary to establish other derived relations in addition to equivalence relations.

Thus, the purpose of the present study was to evaluate whether the go/no‐go procedure with compound stimuli would establish derived asymmetric and transitive relations that require contextual cues (such as comparative, temporal, and deictic relations), thereby extending the evaluation of this procedure to other derived relations beyond equivalence relations.

Consistent with previous research addressing derived comparative relations (e.g., Berens & Hayes, 2007; Dymond & Barnes, 1995; Munnelly et al., 2013, 2019; Whelan et al., 2006), Experiment 1 employed multiple‐exemplar training to establish nonarbitrary relational responding under control of contextual cues. Unlike studies with a MTS procedure that used contextual cues presented separately as contextual stimuli, pictures were presented on colored backgrounds, similarly to Modenesi and Debert's (2015) study with the go/no‐go procedure with compound stimuli. In Experiment 1, background colors served as contextual cues during nonarbitrary relational training. Compound stimuli were two identical figures differing in size displayed side by side on a blue or red background. For the blue background, responses were reinforced only when the left‐side figure was smaller than the one on the right side (e.g., a blue background with a small apple on the left and a large apple on the right). Conversely, for the red background, responses were reinforced only when the left‐side figure was larger than the one on the right side (e.g., red background with a large apple on the left and a small apple on the right). During arbitrary relational training and testing, different equally sized abstract shapes (A1, A2, A3, A4, and A5) replaced the figures to establish arbitrary relational networks. Experiment 2 replicated Experiment 1 without the nonarbitrary training to verify whether asymmetrical and transitive derived relational responding would emerge using the go/no‐go procedure with compound stimuli solely through arbitrary relational training (without explicit size‐based contingencies), as suggested by Alonso‐Álvarez and Pérez‐González (2017, 2018, 2021) for the establishment of derived opposition (symmetrical and nontransitive) relations using the MTS procedure.

## EXPERIMENT 1

### Method

#### Participants

Four undergraduate psychology students participated (three male: P1, P3, and P4 and one female: P2). P1, P2, P3, and P4 were, respectively, 35, 18, 21, and 24 years old. Participants were recruited through in‐class announcements and had no prior familiarity with the experimental analysis of behavior. All the procedures were approved by an ethics committee on human research. Participants were fully debriefed when the experiment concluded. Participants did not receive any monetary or other type of compensation for their participation in the study.

Session duration varied across participants: P1 completed the procedure in a single 60‐min session, P2 required 60‐min sessions for two consecutive days, P3 took 60‐ and 65‐min sessions for two consecutive days, and P4 needed two 60‐min sessions and one 45‐min session for three consecutive days.

### Setting, apparatus, and stimuli

**FIGURE 1:**
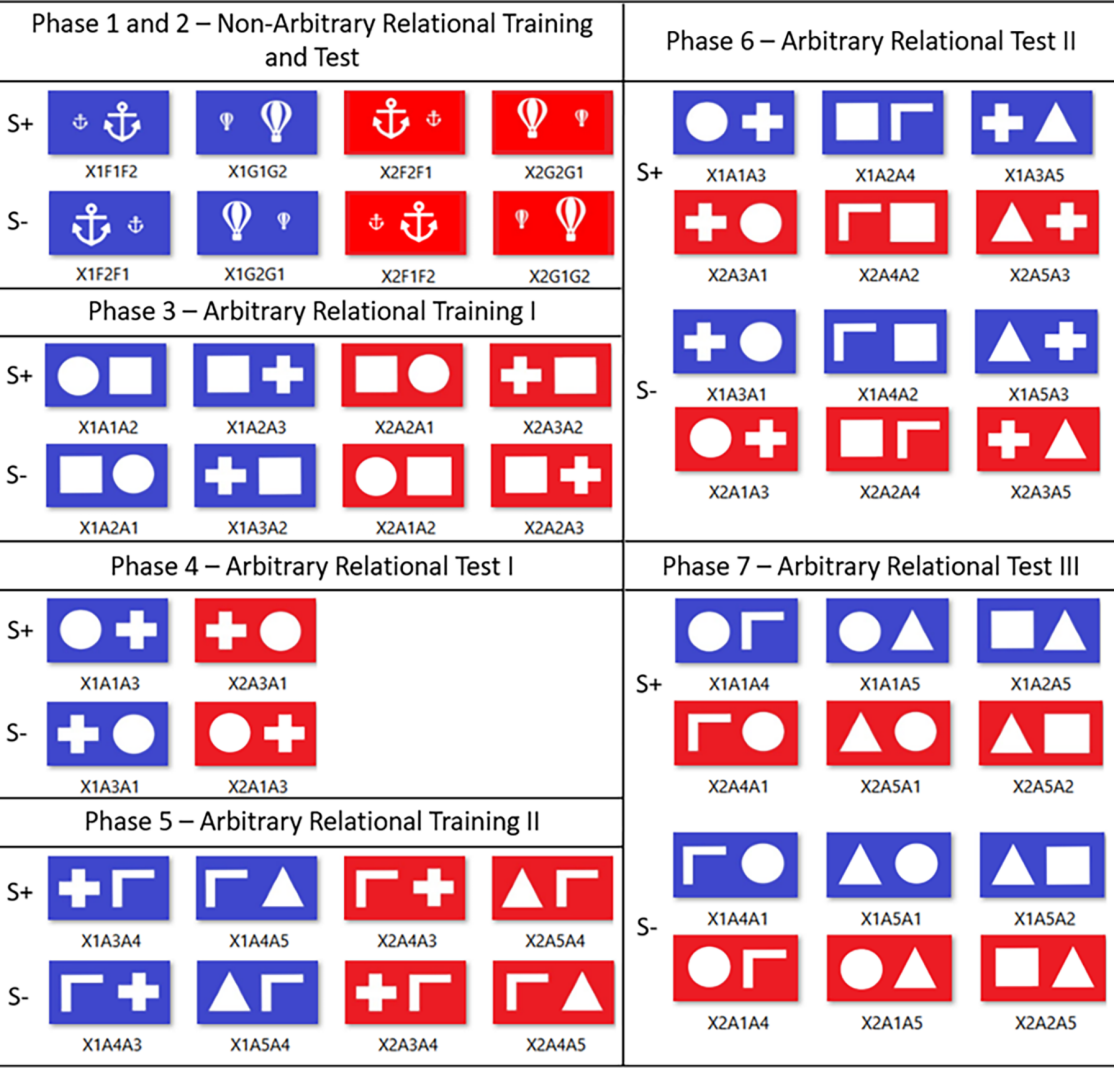
Compound stimuli presented in each phase of the experiment. In Phases 1 and 2, only a few examples of the stimuli used are presented. X1 indicates the blue background and X2 indicates the red background.

**FIGURE 2:**
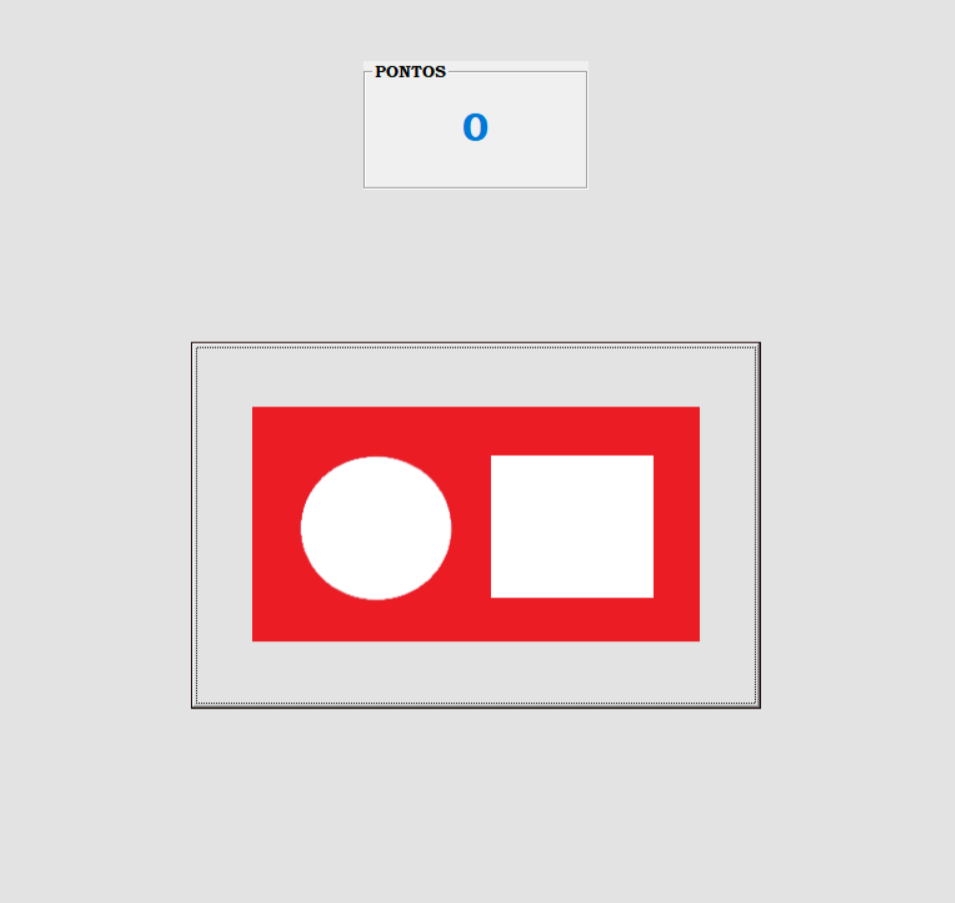
Illustration of the monitor screen during a training trial. The rectangle in the center of the screen displayed the compound stimulus. The point counter is shown above the compound stimulus.

The experiment was conducted individually in a 3‐ x 3‐m room equipped with a computer with the Windows operating system, a mouse, a table, and a chair. Figure 1 shows the stimuli presented on the monitor (1024 x 768 pixels) in each phase. Figure 2 shows an example of a trial from Phase 3 (X2A1A2). Responses were emitted using the mouse. The experimental events were controlled and recorded by a program developed in Visual Basic 6.0.

#### Procedure

The procedure consisted of the following phases:

- Phase 1: Nonarbitrary Relational Training
- Phase 2: Nonarbitrary Relational Test
- Phase 3: Arbitrary Relational Training I
- Phase 4: Arbitrary Relational Test I
- Phase 5: Arbitrary Relational Training II
- Phase 6: Arbitrary Relational Test II
- Phase 7: Arbitrary Relational Test III.

Figure 1 shows all the compound stimuli presented in each of the seven phases.

##### Phase 1: Nonarbitrary Relational Training

In this phase, the go/no‐go procedure with compound stimuli was implemented to establish blue and red backgrounds as the contextual cues for the “larger than” and “smaller than” relations between different sizes of the same picture, such as an airplane, an anchor, and a balloon.

Before the task began, the following instruction was presented on the computer screen:

*This experiment is not an intelligence test and will not assess any intellectual ability. After completing all tasks*, *you will receive an explanation about the purpose of the study. At the beginning of the task*, *you will earn points whenever you click with the mouse in the presence of the correct symbol combinations. Later*, *you may or may not earn points when clicking with the mouse in the presence of correct combinations. The task will gradually become more difficult*, *so please pay close attention*, *even when the task seems very simple*. *Please describe the instructions you have just read to the experimenter. When the experimenter tells you that you may begin*, *click the button labeled “OK” to start the experiment*.

Compound stimuli formed by a background color (e.g., blue or red) and one pair of the same pictures in two different sizes (airplane, alien, microphone, button, anchor, and balloon; Figure 1 presents eight examples) were successively presented in each trial for 4 s, regardless of the participants' responses. Each compound presentation was followed by an intertrial interval of 2 s. The order of presentation of S+ and S‐ compound stimuli varied randomly across trials. The order was restricted such that neither type of trial could be presented more than three times successively. Each block consisted of 24 randomized trials, one for each compound stimulus.

During each trial, participants could click one or many times with the mouse. To be scored as a valid response, the mouse cursor had to be positioned on the stimulus. A differential reinforcement procedure was used to establish clicking with the mouse when the S+ compound stimulus was presented on the screen (e.g., blue background with smaller stimulus on the left and larger on the right or red background with larger stimulus on the left and smaller on the right) and not clicking when the S‐ compound stimulus was presented on the screen (e.g., blue background with larger stimulus on the left and smaller on the right or red background with smaller stimulus on the left and larger on the right; see Figure 1). Each mouse‐clicking response in the presence of the S+ compound stimulus was followed by the addition of 10 points on the counter (see Figure 2) in a continuous reinforcement schedule until the 48th trial. Given that each mouse‐clicking response added 10 points to the counter, participants may have established a response pattern of emitting an initial response and observing the outcome. If the first response generated the consequence, the participant would continue responding within the trial; otherwise, the participant might wait for the trial to end without emitting further responses. To mitigate this effect, starting with Trial 49, responses to the S+ compounds were followed by the consequence on an intermittent schedule of reinforcement (conjunctive fixed ratio [FR] 1 and variable time [VT] 2.5 s, see Debert et al., 2007). Clicking responses to the S‐ compound and not clicking produced no programmed consequences.

The criteria to advance to the next phase were the occurrence of one or more mouse‐clicking responses during each presentation of S+ compounds and no responses during each presentation of S‐ compounds in two consecutive blocks.

##### Phase 2: Nonarbitrary Relational Testing

In this phase, 24 new compound stimuli composed of six new pictures (TV, apple, atom, house, cup, and bell) were presented in the colored backgrounds (red and blue) to evaluate whether the participants would respond discriminatively to the new stimuli under control of the colored backgrounds. Each compound stimulus was presented for 6 s, followed by a 2‐s intertrial interval. This testing phase was conducted without differential programmed consequences to responses. Each block consisted of 24 randomized trials, one for each compound stimulus. The criteria to advance to the next phase were the same as in Phase 1. If a participant failed to meet the criteria, their participation in the study was discontinued. The same criteria and rationale were applied to the subsequent test phases.

Before the test began, the following instruction was presented on the computer screen: “*This is a new phase. Try to respond based on what you learned in the previous phase. You will not know whether you responded to the correct symbol combinations*, *as points will no longer be displayed*.”

The other parameters were the same as they were in the previous phase.

##### Phase 3: Arbitrary Relational Training I

In this phase, similarly to Phase I, a discriminative training procedure was performed. But eight new compound stimuli formed by the combination of three geometric shapes (A1, A2, and A3) and colored backgrounds (blue: X1 and red: X2) were presented to establish blue and red backgrounds as contextual cues for responding or not in the presence of different combinations of two of the geometric shapes. For example, when A1 was presented on the left and A2 on the right with a blue background, responses were followed by points delivery. However, responses were not followed by reinforcers when these stimuli were presented in inverted positions.

Clicking with the mouse in the presence of the S+ compounds A1A2 and A2A3 presented on the blue background (X1‐A1A2 and X1‐A2A3) and A2A1 and A3A2 presented on the red background (X2‐A2A1 and X2‐A3A2) were followed by reinforcers until the 32nd trial (Figure 1). From the 33rd trial onward, these responses were followed by reinforcement oaaan an intermittent schedule (conjunctive FR 1 and VT 2.5 s). Clicking with the mouse in the presence of the S‐ compounds X2‐A1A2, X2‐A2A3, X1‐A2A1, and X1‐A3A2 was not followed by reinforcers (Figure 1).

Each block was composed of eight randomized trials, and the criteria to move to the next phase were correct performances as described in the previous phase, but in four consecutive blocks.

Before the task began, the following instruction was presented on the computer screen:

*Once again*, *at the beginning of the task*, *you will earn points whenever you click the button in the presence of the correct symbol combinations. Later*, *you may or may not earn points when clicking the button in the presence of correct combinations. The task will gradually become more difficult*, *so pay close attention even when it seems very simple. Click “OK” to begin*.

The other parameters were the same as in Phase 1.

##### Phase 4: Arbitrary Relational Test I

This phase was similar to Phase 2, except that new combinations of stimuli from Phase 3 were presented (A1A3 and A3A1) with red and blue backgrounds (X1‐A1A3, X1‐A3A1, X2‐A1A3, and X2‐A3A1; Figure 1) to evaluate whether emergent control by new stimuli combinations would occur. Each block was composed of four trials, one for each compound stimulus. The criteria to advance to the next phase were the same as in the previous phase.

Responding to the X1‐A1A3 and X2‐A3A1 compounds and not responding to the X1‐A3A1 and X2‐A1A3 compounds demonstrated the derived relational responding. Before testing began, the same instruction from Phase 2: Nonarbitrary Relational Testing was presented.

##### Phase 5: Arbitrary Relational Training II

This phase was similar to Phase 3, except new compound stimuli were used. The S+ compounds were X1‐A3A4, X1‐A4A5, X2‐A4A3, and X2‐A5A4, and the S‐ compounds were X2‐A3A4, X2‐A4A5, X1‐A4A3, and X1‐A5A4. The purpose of this phase was to expand the derived relations involving two new stimuli (A4 and A5) to be related to A3, which became related to A1 and A2 in Phase 3.

##### Phase 6: Arbitrary Relational Test II

This phase was similar to Phase 4, except that the compound stimuli were A1A3, A3A1, A2A4, A4A2, A3A5, and A5A3, with red and blue backgrounds (Figure 1). The test evaluated whether expansion of the derived relations involving the new stimuli (A4 and A5) would be demonstrated.

Responding to the compounds X1‐A1A3, X1‐A2A4, X1‐A3A5, X2‐A3A1, X2‐A4A2, and X2‐A5A3 and not responding to the compounds X2‐A1A3, X2‐A2A4, X2‐A3A5, X1‐A3A1, X1‐A4A2, and X1‐A5A3 demonstrated the expansion of the derived relation under the control of the blue and red as contextual cues. Each block comprised 12 trials, one for each compound stimulus.

##### Phase 7: Arbitrary Relational Test III

This phase was similar to the previous phase, except that compound stimuli were A1A4, A4A1, A1A5, A5A1, A2A5, and A5A2 with red and blue backgrounds (Figure 1). This test evaluated whether other derived relations would be demonstrated. Responding to the compounds X1‐A1A4, X1‐A1A5, X1‐A2A5, X2‐A4A1, X2‐A5A1, and X2‐A5A2 and not responding to the compounds X2‐A1A4, X2‐A1A5, X2‐A2A5, X1‐A4A1, X1‐A5A1, and X1‐A5A2 demonstrated the expansion of the derived relations under the control of blue and red contextual cues.

### Results

**FIGURE 3:**
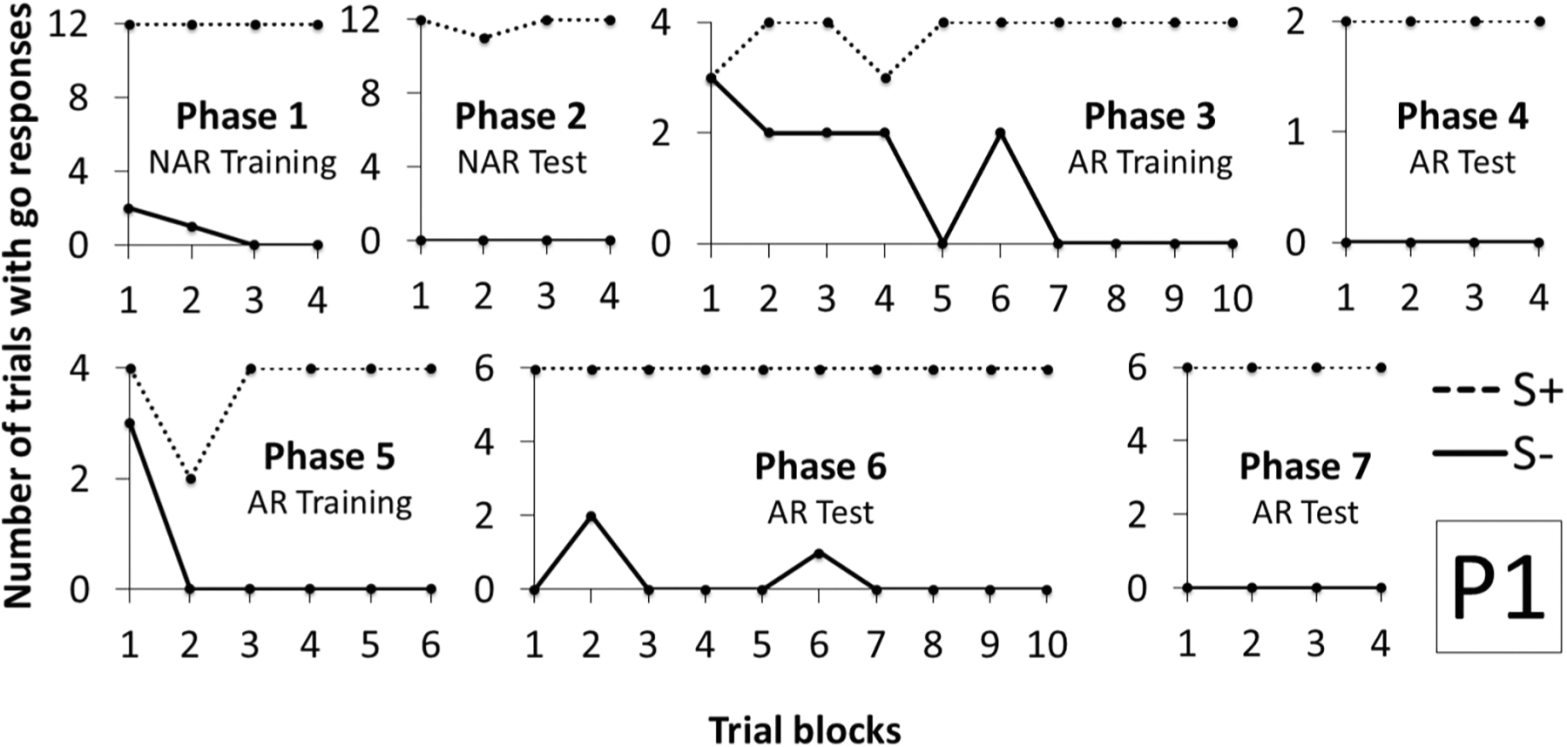
Number of trials with responses during the S+ (dashed line) and S‐ (continuous line) compound stimuli presentation in each block of trials in each phase for P1 in Experiment 1.

Figure 3 shows the number of trials with a response during the S+ and S‐ compound stimulus presentation in each block of trials for Participant 1 (P1) in Experiment 1. P1 needed only four blocks of trials to meet the criteria in Phases 1 and 2. In Phase 3, Arbitrary Relational Training, P1 met the criteria in the 10th block. In Phase 4, Arbitrary Relational Test I, he responded to S+ compounds and did not respond to S‐ compounds in all trials, demonstrating derived control by the contextual cues. In Phase 5, Arbitrary Relational Training II, P1 reached the learning criteria in six blocks, fewer blocks than in Phase 3. In Phase 6, Arbitrary Relational Test II, he responded to all S+ compounds and did not respond to almost all S‐ compounds. In Block 2, he responded incorrectly to X2A2A4 and X1A5A3 compounds, and in Block 6, he responded incorrectly to the X1A4A2 compound. But, after 10 blocks, he reached the criteria and moved to Phase 7, in which other derived relations were demonstrated in just four blocks.

**FIGURE 4:**
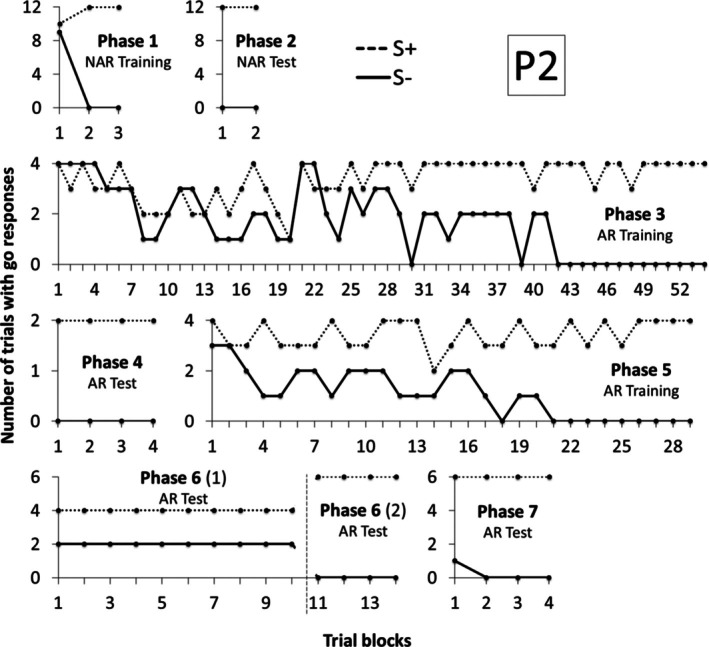
Number of trials with responses during the S+ (dashed line) and S‐ (continuous line) compound stimuli presentation in each block of trials in each phase for P2 in Experiment 1. The number in parentheses next to the phase name shows the session number for that phase whenever it was repeated.

Similarly to P1, P2 needed three and two blocks to reach the criteria, respectively, in Phases 1 and 2 (see Figure 4). In Phase 3, she needed 54 blocks to reach the criteria, more blocks than P1.^1^ In Phase 4, Arbitrary Relational Test I, similarly to P1, he responded correctly to all trials, demonstrating derived control by the contextual cues. In Phase 5 training, she needed 30 blocks to reach the criteria, fewer blocks than in Phase 3. In Phase 6, she responded to the compounds designated as S‐ (X1A3A5, X2A5A3) and did not respond to the compounds designated as S+ (X2A3A5 and X1A5A3) in the first 10 blocks. The software was programmed to interrupt the test at the 10th block if the participants did not meet the criteria. After restarting the software in Phase 6, responding and not responding were reversed, and P2 reached criterion in four blocks. In Phase 7, derived relational responding was also demonstrated across the final blocks, with only one incorrect trial occurring in the first block.

**FIGURE 5:**
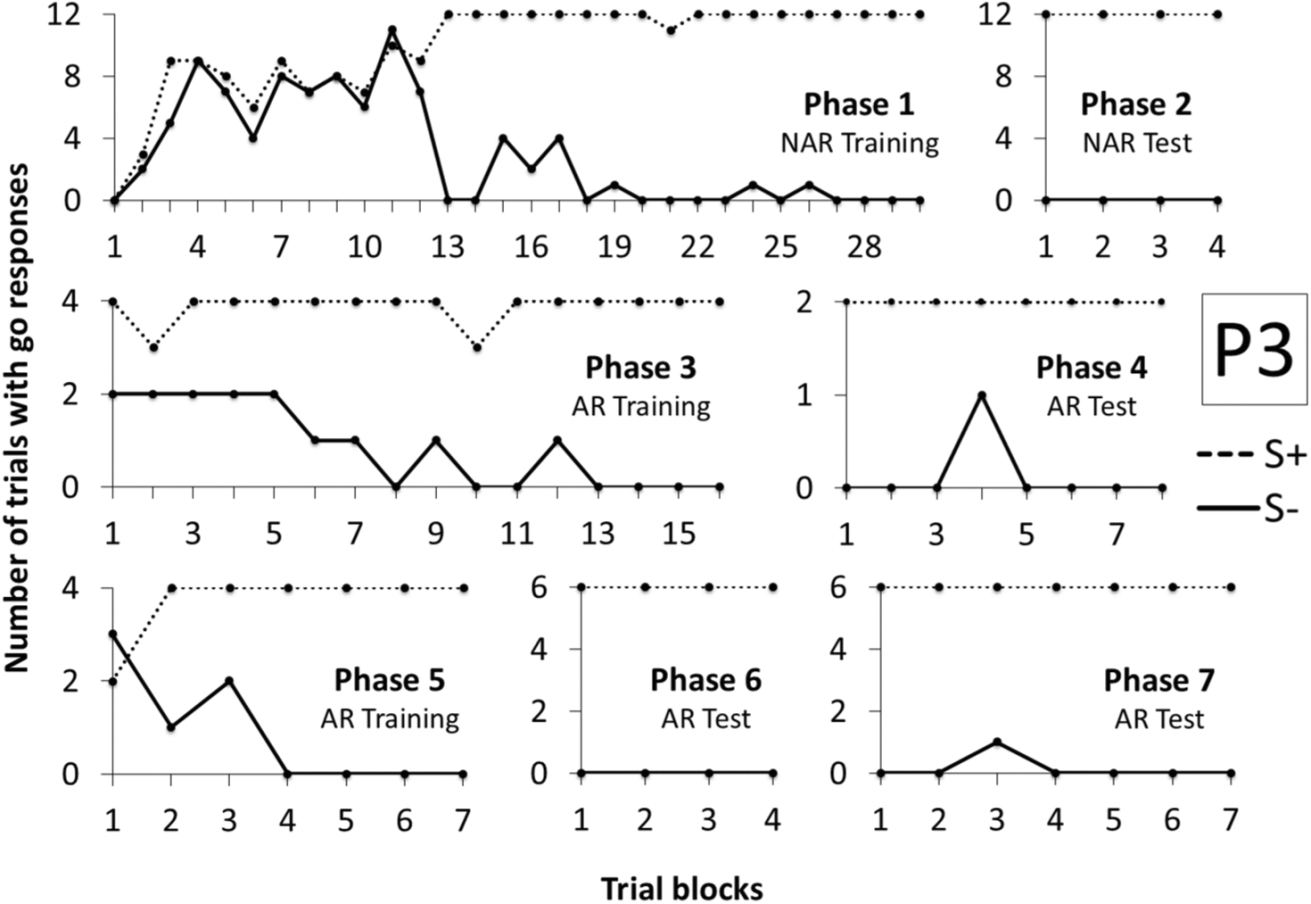
Number of trials with responses during the S+ (dashed line) and S‐ (continuous line) compound stimuli presentation in each block of trials in each phase for P3 in Experiment 1.

Figure 5 shows data for P3. In Phase 1, he met the learning criteria in the 30th block. In Phase 2, he responded correctly in all trials. It is important to highlight that, for this participant, due to a programming error, the learning criterion in Phases 1 and 2 was correct performances in four consecutive blocks (instead of two). In Phase 3, he needed 16 blocks to achieve the learning criterion. In Phase 4, he responded to only one S‐ trial in the 4th block and reached criterion in the 8th block, demonstrating derived control by the contextual cues. In Phase 5, he reached criterion in only seven blocks, fewer blocks than in Phase 3. In Phase 6, he responded correctly in all four blocks, showing derived relational responding in this phase. In Phase 7, he responded incorrectly in just one trial of the third block and reached criterion in the 7th block, indicating derived relational responding comprising the relations tested in Phase 7.

**FIGURE 6:**
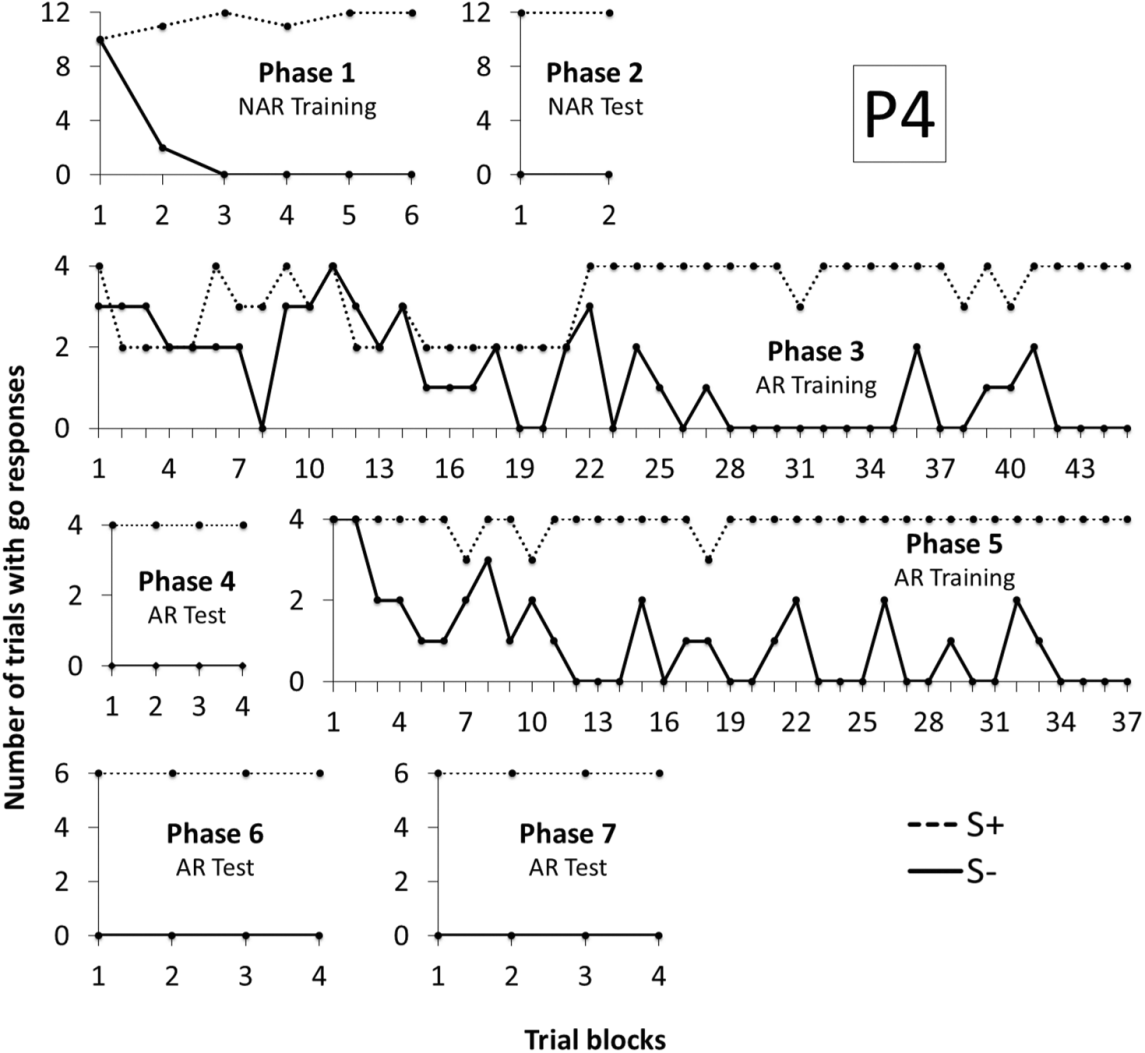
Number of trials with responses during the S+ (dashed line) and S‐ (continuous line) compound stimuli presentation in each block of trials in each phase for P4 in Experiment 1.

Figure 6 shows data for P4. He reached criterion after six blocks in Phase 1 and two blocks in Phase 2. In Phase 3, he reached criterion only after 45 blocks. In Phase 4, accurate performances were exhibited in the fourth block. In Phase 5, he needed 37 blocks to meet criterion. In Phases 6 and 7, he reached criterion in the first four consecutive blocks, demonstrating the emergence of the derived relations.

### Discussion

The purpose of Experiment 1 was to verify whether the go/no‐go procedure with compound stimuli would establish asymmetric and transitive relations (e.g., comparative relations), thereby extending the evaluation of this procedure to derived relational responding beyond equivalence relations.

All participants met the learning criteria in the Nonarbitrary Relational Training and Testing phases, a performance expected for adults with an advanced verbal repertoire (e.g., Dymond & Barnes, 1995; Munnelly et al., 2013; Murphy & Barnes‐Holmes, 2010; O'Hora et al., 2002; Perez et al., 2017; Steele & Hayes, 1991; Whelan et al., 2006).

In Arbitrary Relational Training I, participants required from 40 to 216 trials to meet the criteria, similar to other studies that established asymmetric and transitive relations (e.g., Dymond & Barnes, 1995; Munnelly et al., 2013; Perez et al., 2017). In Arbitrary Relational Training II, all participants met the criteria in fewer blocks than in the previous training. Derived relations were demonstrated for almost all participants in the first blocks of Phases 4, 6, and 7 (Arbitrary Relational Tests I, II, and III), except for P2, who showed derived relations only after repeating tests in Phase 6.

These results indicate that the go/no‐go procedure with compound stimuli was effective to establish derived asymmetric and transitive relations between the stimuli, similarly to studies that used other procedures, such as the MTS and relational evaluation procedures (e.g., Barnes‐Holmes et al., 2004; Belisle et al., 2020; Berens & Hayes, 2007; Dymond & Barnes, 1995; Gorham et al., 2009; Luoma et al., 2024; Munnelly et al., 2013, 2019; O'Hara et al., 2002; Perez et al., 2017; Reilly et al., 2005; Roche & Dymond, 2008; Whelan et al., 2006; Vitale et al., 2008). Considering that the go/no‐go procedure with compound stimuli has the advantage of simplicity in the stimuli displays, it can be used in applied settings to establish different kinds of complex derived relational responding, avoiding so‐called stimulus overselectivity or restricted stimulus control (Dube & McIlvane, 1999; Schreibman et al., 1982).

Furthermore, the results of Experiment 1 raise questions regarding the relevant controlling variables for the establishment of this type of complex operant. According to Hayes et al. (2001), the control exerted by the contextual cues over the relations between some physical dimension (e.g., size) of the stimuli during the nonarbitrary relational training would account for the derived performance observed in the tests with new arbitrary relations. In other words, if new arbitrarily derived relations were demonstrated, contextual control over relations based on some physical dimension of stimuli established during nonarbitrary training must be in effect if new arbitrarily derived relations were demonstrated (e.g., Dymond & Barnes, 1995; Hayes et al., 2001; Munnelly et al., 2013; Perez et al., 2017; Whelan et al., 2006).

In Experiment 1 of the present study, an additional subset of variables common to both nonarbitrary and arbitrary relational training should be analyzed: the background color serving as context controlling responding to the relative positions of stimuli within compound configurations. For example, in nonarbitrary relational training, responses to the compound stimulus with the smaller image positioned on the left were reinforced when the background was blue and extinguished when it was red. Conversely, responses to compounds with the larger picture positioned on the left were reinforced when the background was red and extinguished when it was blue. A similar contingency was presented during arbitrary relational training: Responses to A1‐left/A2‐right compounds were exclusively reinforced when the background was blue, and responses to A2‐left/A1‐right compounds were exclusively reinforced when the background was red. The results demonstrate contextual control over relational responding that could be based on stimulus position and the background color serving as the discriminative contextual cue for both nonarbitrary and arbitrary relations (see Debert et al., 2009, for conditional control by position of the elements of compound stimuli and Green et al., 1993, for sequences or ordering relations). On this basis, one may ask whether nonarbitrary training is necessary for establishing derived relational responding characterized as asymmetrical and transitive (e.g., comparative relations). Alonso‐Álvarez and Pérez‐González (2017, 2018, 2021) produced derived opposition relations (i.e., symmetrical and nontransitive) without the need for nonarbitrary relational training and testing, requiring only the establishment of contextual control over equivalence classes and exclusion‐based responding. Thus, to assess whether nonarbitrary relational training was necessary to produce the observed asymmetrical and transitive derived performance with the go/no‐go procedure with compound stimuli, Experiment 2 was conducted in which arbitrary training was implemented without the nonarbitrary training and testing phases.

## EXPERIMENT 2

### Method

#### Participants

Three undergraduate psychology students participated (two males, P5 and P7, and one female, P6). P5 and P7 were 21 years old, and P6 was 25 years old. Participants were recruited through in‐class announcements and had no prior familiarity with the experimental analysis of behavior. All the procedures were approved by an ethics committee on human research, and the participants were fully debriefed when the experiment concluded.

Session duration varied across participants: P5 required one 45‐min session, P6 completed the procedure in two sessions (50 and 40 min) on consecutive days, and P7 completed the procedure after 3 days of sessions (70, 50, and 40 min).

#### Setting, apparatus, and stimuli

The setting, apparatus, and stimuli were the same as those described in Experiment 1, except that the stimuli from Phase 1 were not used.

#### Procedure

The procedure consisted of the following phases:

- Phase 1: Arbitrary Relational Training
- Phase 2: Arbitrary Relational Test
- Phase 3: Arbitrary Relational Training II
- Phase 4: Arbitrary Relational Training III
- Phase 5: Arbitrary Relational Test II

##### Phase 1: Arbitrary Relational Training

This phase was similar to Phase 3 in Experiment 1. But, differently from Experiment 1, Experiment 2 adopted a three‐step training process to produce faster acquisition of the training relations, given that P2 and P4 from Experiment 1 required an extensive number of trials (respectively, 432 and 360 trials) to meet criterion.

Thus, this phase was divided into three steps: Phase 1.1, Phase 1.2, and Phase 1.3. In Phase 1.1, only the compound stimuli with the blue background were presented (related compounds: X1‐A1A2 and X1‐A2A3; unrelated compounds: X1‐A2A1 and X1‐A3A2) in each block. In Phase 1.2, only the compound stimuli with the red background were presented (related compounds: X2‐A2A1 and X2‐A3A2; unrelated compounds: X2‐A1A2 and X2‐A2A3) in each block. Each block in Phases 1.1 and 1.2 contained four trials. In Phase 1.3, all compound stimuli (with blue and red backgrounds) were presented in each 8‐trial block. All other parameters were the same as those described in Phase 3 from Experiment 1.

##### Phase 2: Arbitrary Relational Test I

This phase was identical to Phase 4 from Experiment 1.

##### Phase 3: Arbitrary Relational Training II

This phase was the same as Phase 5 from Experiment 1, except that stimulus A5 was not presented in order to have fewer training trials. The following compounds were presented: X1‐A3A4 and X2‐A4A3 (S+) and X2‐A3A4 and X1‐A4A3 (S‐).

##### Phase 4: Arbitrary Relational Training III

In this phase, all compound stimuli presented during the training phases (Phase 1 and Phase 3) were presented. Each block comprised 12 trials, and all other parameters were identical to those used in Phase 1.

##### Phase 5: Arbitrary Relational Test II

This phase was similar to Phase 2, except for the compound stimuli that were presented. Responding to the compounds X1‐A1A3, X1‐A2A4, X1‐A1A4, X2‐A3A1, X2‐A4A2, and X2‐A4A1 and not responding to the compounds X2‐A1A3, X2‐A2A4, X2‐A1A4, X1‐A3A1, X1‐A4A2, and X1‐A4A1 demonstrated the derived performances. The other parameters were the same as those described in Phase 6 from Experiment 1.

### Results

**FIGURE 7:**
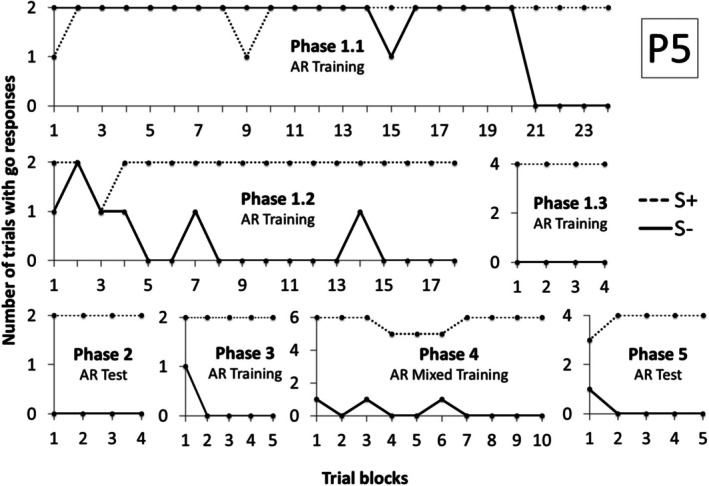
Number of trials with responses during the S+ (dashed line) and S‐ (continuous line) compound stimuli presentation in each block of trials in each phase for P5 in Experiment 2.

Figure 7 shows the number of trials with a go response during the S+ and S‐ compound stimuli presentation in each block of trials for P5 in Experiment 2. To meet criterion, P5 required 24 blocks in Phase 1.1, 18 blocks in Phase 1.2, and 4 blocks in Phase 1.3. Accurate performances were demonstrated in Phase 2 (Arbitrary Relational Test). In Phase 3 (Arbitrary Relational Training II) and Phase 4 (with compound stimuli from Phases 1 and 3), P5 required, respectively, 4 and 10 blocks to reach criterion. During Phase 5 (Arbitrary Relational Test II), P5 emitted two incorrect responses in the first block but achieved 100% accuracy across the remaining four blocks.

**FIGURE 8:**
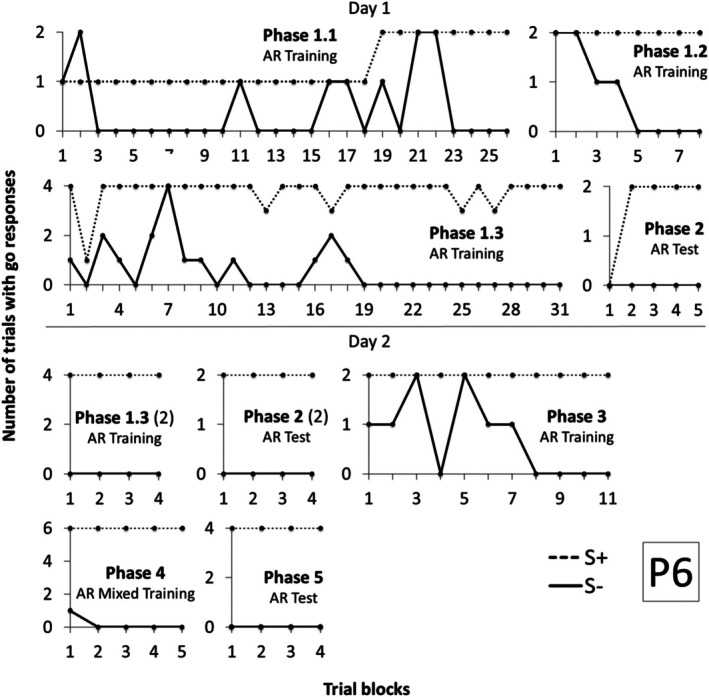
Number of trials with responses during the S+ (dashed line) and S‐ (continuous line) compound stimuli presentation in each block of trials in each phase for P6 in Experiment 2. The number in parentheses next to the phase name shows the session number for that phase whenever it was repeated.

Participant 6 (P6) met criterion after 25 blocks during Phase 1.1, 8 blocks in Phase 1.2, and 31 blocks in Phase 1.3 (Figure 8). During the Arbitrary Relational Test I (Phase 2), no responses were emitted in the first block. However, 100% accuracy was observed across the subsequent four blocks. Before initiating Phase 3, P6 requested the interruption of the procedure because she was tired. On the subsequent day (Day 2), Phases 1.3 and 2 were conducted again before moving on to Phase 3. P6 showed accurate performances in 4 blocks for Phases 1.3 and 2. In Phases 3 and 4, P6 required, respectively, 11 and 5 blocks to meet criterion. During Phase 5 (Arbitrary Relational Test II), performance was 100% accurate in just 4 blocks.

**FIGURE 9:**
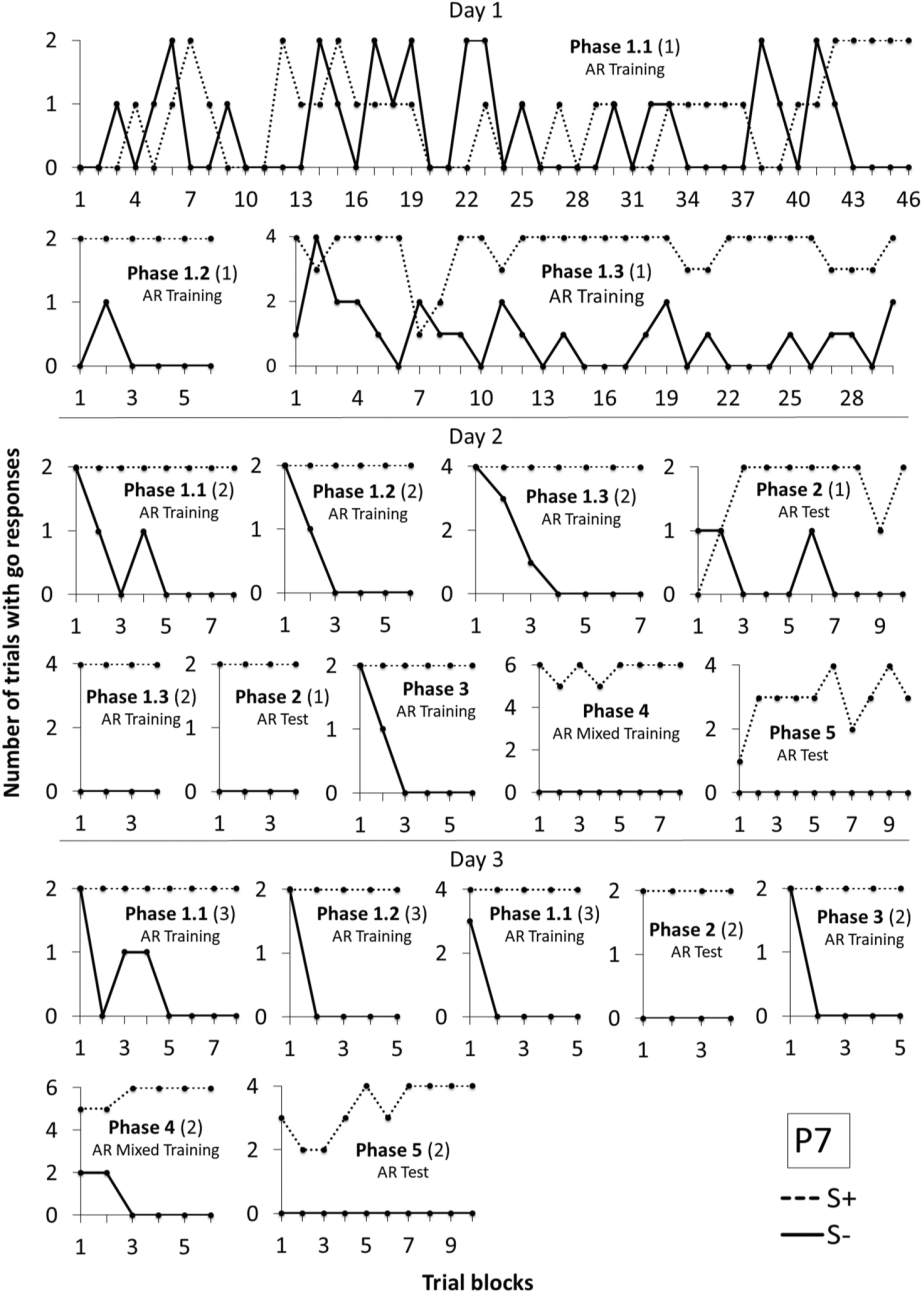
Number of trials with responses during the S+ (dashed line) and S‐ (continuous line) compound stimuli presentation in each block of trials in each phase for P7 in Experiment 2. The number in parentheses next to the phase name shows the session number for that phase whenever it was repeated.

Participant 7 (P7) required more training blocks to meet criterion (Figure 9) in Phase 1. On the first day, P7 met criterion after 46 blocks in Phase 1.1 and 6 blocks in Phase 1.2. In Phase 1.3 (mixed training), P7 did not reach criterion after 30 blocks, and the procedure was interrupted because 70 min had elapsed.

On the next day, P7 repeated Phases 1.1 and 1.2 and reached criterion after, respectively, 8 and 6 blocks. In Phase 1.3, he met criterion after 7 blocks but failed to meet criterion in Phase 2 after 10 blocks of trials. Considering that, Phase 1.3 was again repeated, and criteria were reached in 4 blocks. He reached criterion in Phases 2, 3, and 4 after, respectively, 4, 6, and 8 blocks. In Phase 5, he did not reach criterion even after 10 blocks, and the procedure was interrupted because the participant requested it after 50 min.

On the next day, P7 repeated Phases 1.3 and 2 and reached criterion after 4 blocks in each of these phases. Afterward, Phases 3, 4, and 5 were repeated, and P7 met criterion, respectively, in 5, 6, and 10 sessions, suggesting that derived relational responding was produced after retraining.

### Discussion

Experiment 2 evaluated whether the nonarbitrary relational training used in Experiment 1 was necessary to establish derived asymmetric and transitive relations using the go/no‐go procedure with compound stimuli. Results from Experiment 2 showed that derived asymmetric and transitive relations emerged without nonarbitrary relational training. Therefore, in contrast to Hayes et al. (2001), establishing control over relations based on some physical dimension (e.g., size) of stimuli through nonarbitrary training was not necessary for derived arbitrary relations to emerge.

These findings, produced by the go/no‐go procedure with compound stimuli for derived asymmetric and transitive relations, are in line with the alternative account proposed by Alonso‐Álvarez and Pérez‐González (2017, 2018, 2021) for derived opposition relations produced by the MTS procedure. Alonso‐Álvarez and Pérez‐González (2021) proposed that nonarbitrary relational training and testing would be unnecessary and that the derived relational responding would require only the establishment of contextual control over equivalence classes and exclusion‐based responding. Specifically in the case of the asymmetric and transitive relations established in the present study, the contextual control over relational responding could be based on stimulus position, and the background color would serve as the discriminative contextual cue for derived arbitrary relations (see Debert et al., 2009, for conditional control by position of the elements of compound; see Green et al., 1993, for sequences or ordering relations). Thus, it can be hypothesized that contextual control over the positions of stimuli in arbitrary relational training alone would suffice to generate derived asymmetry and transitivity relations.

Although derived relational responding was produced without nonarbitrary relational training, it should be noted that two participants from Experiment 2 required further retraining. In contrast, no participants from Experiment 1 required retraining. Thus, it is possible that nonarbitrary relational training would improve both training and derived performance. However, it is also important to consider that the arbitrary relational training differed between the two experiments. In Experiment 1, this training was conducted with all stimulus combinations. In contrast, in Experiment 2, the training was divided into three phases (or steps): The first phase used only stimuli with a blue background color, the second phase used only those with a red background, and the third phase included all possible combinations, similar to Experiment 1. A comparative analysis was conducted between the arbitrary relational training data obtained in Experiment 1 and the modified procedure used in Experiment 2. It is important to note that Experiment 1 included a nonarbitrary relational training phase, which may have facilitated subsequent arbitrary relational training acquisition. In contrast, Experiment 2 employed a procedural modification—the division of training into three distinct phases—specifically directed at facilitating acquisition given the absence of nonarbitrary relational training. The results show that the mean number of trials to reach the arbitrary relational training criterion was comparable across the studies (*M* = 31.25 in Experiment 1 vs. *M* = 32.00 in Experiment 2). However, variability of performance, as measured by the standard deviation, was substantially reduced in Experiment 2 (*SD* = 12.17) relative to Experiment 1 (*SD* = 21.53). This suggests that the training phase in Experiment 2 effectively facilitated acquisition and successfully compensated for the absence of the preceding nonarbitrary relational training. Further studies could investigate whether performances would be similar if the same phases were implemented with and without nonarbitrary training.

## GENERAL DISCUSSION

Results of Experiments 1 and 2 demonstrated that the go/no‐go procedure with compound stimuli can be used to establish derived asymmetric and transitive relations. All participants from both experiments met the learning criteria in the training phases and showed derived performances in the tests. Notably, the results from both experiments demonstrated that this procedure was effective in producing these relations in the same manner as other procedures including successive go/no‐go (e.g., Brassil et al., 2019), the yes/no procedure (e.g., Sbrocco et al., 2025), the implicit relational assessment procedure (for a review, see Barnes‐Holmes & Harte, 2022), and the relational evaluation procedure (e.g., Munnelly et al., 2013). This suggests that the derived repertoires can emerge under different circumstances.

Debert et al. (2007) suggested that with the go/no‐go procedure, the stimuli to be observed are closely spaced or presented as figure–ground (e.g., Debert et al., 2009; Modenesi & Debert, 2015) and thus can be scanned with little or no change in the direction of gaze, thus more closely resembling real‐life experiences (e.g., Munnelly et al., 2013). Therefore, the go/no‐go procedure with compound stimuli may benefit populations who present restricted stimulus control (e.g., Bourret et al., 2012; Da Hora et al., 2018; Stromer et al., 1993).

Compared with Experiment 1, the results from Experiment 2 indicated that derived asymmetric and transitive performances would emerge without the nonarbitrary relational training that was considered necessary to establish derived arbitrary relations (e.g., Hayes et al., 2001). Alonso‐Álvarez and Pérez‐González (2017, 2018, 2021) also suggest that nonarbitrary relational training may not be required to produce the derived relational responding in adults with well‐developed verbal repertories. They have argued that opposition relations (typically classified as symmetric and nontransitive) may instead reflect contextual control over equivalence classes and exclusion responding (i.e., in a MTS trial, responding to the comparison stimulus that is not a member of an established equivalence class). Thus, the prerequisites under which the derived relational responding is produced are yet to be determined, at least in those experimental contexts. However, it is important to consider that these studies were conducted with adult participants who possessed a well‐developed verbal repertoire. Thus, the hypothesis that these performances reflect a higher order operant whose foundations are established very early in the development of individuals' verbal repertoires cannot be ruled out. Future studies could replicate these experiments with participants with less developed verbal repertoires, such as children (e.g., Berens & Hayes, 2007) or neurodivergent individuals (e.g., Belisle et al., 2020).

The present results raise another question: Could asymmetric/transitive relations (such as comparative, temporal, or deictic relations) similarly emerge from contextual control over relative stimulus positions or ordinal relations? In this case, the minimal sufficient condition for establishing derived asymmetric/transitive relations could be contextual control over positional or ordinal relations established during the arbitrary relational training (see Debert et al., 2009, for conditional control by position of the elements of compound stimuli and Green et al., 1993, for control by sequences or ordinal relations). Considering contextual control over relative position or ordinal relations, the blue background used in the current study would establish the stimulus position (i.e., left) or sequences in one direction (i.e., A1‐A2‐A3‐A4‐A5), whereas the red background would establish the opposite stimulus position (i.e., right) or sequence direction (i.e., A5‐A4‐A3‐A2‐A1). Future studies could evaluate the relation between position and/or order relations and derived comparative relations. As suggested by Debert et al. (2009), the go/no‐go procedure has some advantages over the MTS procedure for the study of emergent stimulus–stimulus relations when training involves relative position relations considering that one cannot separate the constituent elements of stimulus‐position compound relation into sample and comparison stimuli.

Future studies could extend the current evaluation by conducting transformation‐of‐function tests after training and testing with the go/no‐go procedure with compound stimuli. After establishing a comparative relational network (A1 < A2 < A3 < A4 < A5) with the go/no‐go procedure with compound stimuli, participants would be trained to press the space bar in the presence of one of the stimuli (for example, A3) with a specific frequency. After this training, all the remaining stimuli would be successively presented and participants should be asked to press the space bar more or less frequently. Responding more frequently to stimuli A4 and A5 than to A1 and A2 would demonstrate that comparative (more than/less than) relations were established.

In conclusion, the present findings extend the empirical evidence that asymmetric and transitive derived relational responding can be produced by the go/no‐go procedure with compound stimuli and raise important questions regarding the training prerequisites to establish derived relational responding. Advancing empirical knowledge addressing these prerequisites may contribute to the refinement of teaching technologies to establish complex derived relational repertoire in applied settings.

## AUTHOR CONTRIBUTIONS

**Rafael Diego Modenesi:** conceptualization, methodology, software, data curation, investigation, validation, formal analysis, funding acquisition, project administration, writing ‐ original draft. **Paula Debert:** conceptualization, formal analysis, supervision, funding acquisition, visualization, resources, writing ‐ review and editing.

## CONFLICT OF INTEREST STATEMENT

We have no known conflict of interest to disclose.

## ETHICS APPROVAL

All the procedures were approved by an ethics committee on human research of Universidade de Sao Paulo (CAAE: 37239019.7.0000.5561). Participants were fully debriefed when the experiment concluded, and they did not receive any monetary or other type of compensation for their participation in the study.

## Data Availability

Data are available on request.

## Footnotes

^1^
The software was programmed to interrupt the training in every 10th block if the participants did not meet the criteria or after four blocks with 100% accuracy. After restarting the software in Block 51, the participant met the learning criteria in the next four blocks.
